# Exploring the Role of Social Media Use Motives, Psychological Well-Being, Self-Esteem, and Affect in Problematic Social Media Use

**DOI:** 10.3389/fpsyg.2020.617140

**Published:** 2020-12-16

**Authors:** Bruno Schivinski, Magdalena Brzozowska-Woś, Ellena Stansbury, Jason Satel, Christian Montag, Halley M. Pontes

**Affiliations:** ^1^School of Media and Communication, RMIT University, Melbourne, VIC, Australia; ^2^The International Cyberpsychology and Addictions Research Laboratory (iCARL), University of Tasmania, Launceston, TAS, Australia; ^3^Department of Marketing, Gdansk University of Technology, Gdańsk, Poland; ^4^Psychology Department, Nottingham Trent University, Nottingham, United Kingdom; ^5^School of Psychological Sciences, University of Tasmania, Launceston, TAS, Australia; ^6^Department of Molecular Psychology, Institute of Psychology and Education, Ulm University, Ulm, Germany

**Keywords:** problematic social media use, social media motives, problematic behavior, well-being, self-esteem, affect, problematic consumer behavior

## Abstract

Given recent advances in technology, connectivity, and the popularity of social media platforms, recent literature has devoted great attention to problematic Facebook use. However, exploring the potential predictors of problematic social media use beyond Facebook use has become paramount given the increasing popularity of multiple alternative platforms. In this study, a sample of 584 social media users (*M*_age_ = 32.28 years; 67.81% female) was recruited to complete an online survey assessing sociodemographic characteristics, patterns, and preferences of social media use, problematic social media use (PSMU), social media use motives, psychological well-being, self-esteem, and positive and negative affect. Results indicated that 6.68% (*n* = 39) of all respondents could be potentially classed as problematic users. Moreover, further analysis indicated that intrapersonal motive (β = 0.38), negative affect (β = 0.22), daily social media use (β = 0.18), surveillance motive (β = 0.12), and positive affect (β = −0.09) each predicted PSMU. These variables accounted for about 37% of the total variance in PSMU, with intrapersonal motive driving the greatest predictive contribution, over and above the effects of patterns of social media use and sociodemographic variables. These findings contribute to the increasing literature on PSMU. The results of this study are discussed in light of the existing literature on PSMU.

## Introduction

The use of social media has been growing exponentially, with social media being a globally recognized tool used not only by individuals, but by organizations, brands, and celebrities globally. In this study, social media is defined as “web-based services that allow individuals to (1) construct a public or semi-public profile within a bounded system, (2) articulate a list of other users with whom they share a connection, and (3) view and traverse their list of connections and those made by others within the system” (Boyd and Ellison, 2007). Examples of popular social media platforms include Facebook, Instagram, YouTube, Twitter, and LinkedIn, among others.

Social media reaches a large number of users worldwide, with as many as 71% of adolescents accessing more than one platform, with about 24% of all adolescents admitting to being constantly online due to increased mobile accessibility via smartphones (Lenhart et al., 2015). Social media use is not specifically limited to adolescents as adults also use social media platforms as an important socializing and information tool (Heo et al., 2015; Schivinski et al., 2019), with social media use influencing different types of behavior across the entire lifespan (Kuss et al., 2013; Schivinski, 2019). Judicious use of social media leads to many advantageous and beneficial psychosocial outcomes, such as greater social support (Tifferet, 2020), increased friendship quality (Wang et al., 2019b), higher levels of happiness (Ward et al., 2018), and decreased depression levels (Wang et al., 2019a).

However, although moderate use of social media does not interfere with overall functioning and psychological well-being (Twigg et al., 2020), negative effects stemming from social media use have been examined in the context of excessive and problematic usage. Research investigating the negative outcomes of social media use has indicated that problematic social media use (PSMU) may lead to deteriorated psychological well-being (Huang, 2012; Lin et al., 2016) and overall health (Pontes, 2017; Brailovskaia et al., 2020). One of the most prominent negative effects of social media use relates to problematic use stemming from dysregulated usage (Kuss and Griffiths, 2017; Radovic et al., 2017), often triggered by the design of the social media platforms and the prevailing data business model (Montag et al., 2019). However, the literature remains inconclusive, fragmented, and heavily skewed toward the investigation of a single, specific social media platform (e.g., Facebook) (e.g., see Pontes et al., 2016; da Veiga et al., 2019; Rozgonjuk et al., 2020a,b; Sindermann et al., 2020; for exceptions Marengo et al., 2020).

Despite not being an officially recognized mental health disorder, previous research has demonstrated that PSMU can longitudinally influence a wide range of psychiatric outcomes and behaviors, including, but not limited to, increased severity of insomnia, stress, depression, and anxiety (Brailovskaia and Margraf, 2017; Brailovskaia et al., 2019a), in addition to suicide-related outcomes (Brailovskaia et al., 2020). At the cross-sectional level, PSMU has been linked to decreased sleep quality and life satisfaction (Sha et al., 2019; Buda et al., 2020; Duradoni et al., 2020), emotional maladjustment (Hawes et al., 2020), lower productivity (Rozgonjuk et al., 2020d), narcissistic traits (Brailovskaia et al., 2020), poorer psychological functioning (Sampasa-Kanyinga and Lewis, 2015), phubbing (Karadag et al., 2015), binge drinking (Spilkov et al., 2017), and addictive usage (Andreassen and Pallesen, 2014; Ryan et al., 2014; Andreassen, 2015; Pontes et al., 2018).

Early epidemiological research including representative samples reported PSMU prevalence rates of about 4.0% among adolescents in Germany (Müller et al., 2016), 4.5% in Hungarian adolescents (Bányai et al., 2017), 2.9% in the general Belgian population (De Cock et al., 2014), and 4.9% in the British population (Pontes et al., 2018). More recent epidemiological research has reported a 1-year PSMU prevalence of 2.6% among adolescents and younger adults (Wartberg et al., 2020) from the German population (Reer et al., 2020).

Based on these recent developments, the present study aims to contribute to the growing body of knowledge on social media use by exploring the role of key factors accounting for PSMU more broadly (i.e., without being attached to a specific social media platform). Given the relatively high number of studies that have investigated problematic Facebook use specifically, rather than using a broader definition of PSMU, understanding potential contributing factors to broad PSMU is paramount because of the continuous and steady growth of social media users and platforms worldwide. Furthermore, this approach is in agreement with previous literature suggesting that PSMU should be investigated more broadly, without association to specific social media platforms (Griffiths, 2012).

Understanding PSMU risk factors is key to advancing research on social media behavior. In this context, motives for social media use have been suggested as a key correlate of PSMU (Wang et al., 2016). Previous research has shown that individuals high on affiliative tendency and communication competence often use social media to expand their social networks, demonstrating lower levels of self-focus and intrapersonal social media use motives (Lee and Kim, 2014). Similarly, previous research has established that the desire to maintain social relationships among social media users is associated with life satisfaction (Rae and Lonborg, 2015). Psychological well-being, self-esteem, and affect have also been established as key correlates of PSMU, based on the previously established cognitive-behavioral model of pathological internet use (PIU) (Davis, 2001).

Research on PIU has also revealed significant relationships between internet use and the management of emotional difficulties and negative life outcomes (Caplan, 2002, 2010). This may imply that PIU, generally, and PSMU, more specifically, may emerge as dysfunctional coping mechanisms to help users manage their negative emotional and affective states (Kardefelt-Winther, 2014). Thus, people who use social media to help cope with adverse outcomes and existing difficulties (e.g., poor psychological well-being), may feel compelled to systematically use social media on a frequent basis (Radovic et al., 2017), rendering them potentially more vulnerable to developing PSMU due to increased duration of, and exposure to, social media (Brailovskaia et al., 2019b).

In order to help further advance current understanding of key PSMU risk factors, the aim of this study is to explore and empirically examine the role of key psychosocial predictors on PSMU, while accounting for potential confounding effects stemming from frequency of social media use and sociodemographic factors.

## Method

### Participants and Procedures

A cross-sectional study using an online survey was carried out to explore the role of motives for using social media, positive and negative affect, psychological well-being, and self-esteem in predicting PSMU in a convenience sample of English-speakers 759 social media users. An online survey hosted on Qualtrics was created and advertised from May to June 2017 on different social media platforms that included Facebook, Twitter, Instagram, and Snapchat where no form of incentives (e.g., financial) were offered to potential participants. In the online survey, social media use was defined to all potential participants in terms of the following social media platforms: “*Facebook (including Messenger)*”, “*Twitter*,” “*Instagram*,” “*Snapchat*,” and “*Others*” to ensure that the study would capture broad social media behavior. The online survey was brief and expected to be completed in ~15 min on average.

All potential participants were informed about the nature of the study and required to provide online informed consent in order to partake in the study. Participants were provided with all the necessary information about the ethical aspects of this study (e.g., anonymity, confidentiality, right to discontinue participation, right to withdraw their data after completing the study). All participants were required to be aged at least 18 years old in order to be eligible to partake in the study. The present study has been granted ethical approval [*with the Nottingham Trent University*] Ethics Committee.

A total of 175 participants were excluded from analyses due to missing data, resulting in a final sample of 584 participants (*M*_age_ = 32.28 years, *SD* = 12.62 years), of which 67.81% (*n* = 396) were female. About 22.88% (*n* = 157) of the participants reported not being in a relationship. In terms of social media use, 96.58% (*n* = 564) reported accessing social media through a smartphone, and Facebook was reported to be the most used social media platform (55.82%, *n* = 326), followed by Instagram (20.03%, *n* = 117), with other platforms (e.g., Tumblr, Reddit) accounting for 3.91% (*n* = 23) of the responses. Furthermore, the majority of participants declared using only one social media platform (38.86%, *n* = 227) and about 34.76% (*n* = 203) of all participants reported accessing social media very frequently (i.e., on a daily basis). Finally, 6.68% (*n* = 39) of all participants could be classified as problematic users (see Table 1).

**Table 1:** Structure of the sample, social media (SM) patterns of use and preferences, and descriptive statistics (*n* = 584).

**Variable**	
Gender (female, %)	396 (67.81)
Age (years) (*mean, SD*)	32.28 (12.62)
Not in a relationship (*n*, %)	157 (26.88)
Most used SM platform (*n*, %)	
Facebook (including “Messenger”)	326 (55.82)
Instagram	117 (20.03)
Twitter	60 (10.27)
Snapchat	58 (9.93)
Other	23 (3.91)
**Number Of SM Platforms Used Daily, (*****n*****, %)**	
1	227 (38.86)
2	133 (22.77)
3	131 (22.43)
4	69 (11.81)
5	19 (3.25)
6 or more	5 (0.85)
**Daily SM Use, (*****n*****, %)**	
Very rarely	24 (4.11)
Rarely	88 (15.07)
Frequently	155 (26.54)
Very frequently	203 (34.76)
Access to SM via smartphone (*n*, %)	564 (96.58)
Daily SM occurrences (*mean, SD*)	18.58 (63.30)
Minutes spent per each session (*mean, SD*)	5.58 (38.76)
Problematic SM use (*n*, %)	
Non-problematic SM use	545 (93.32)
Problematic SM use	39 (6.68)
**Descriptive Statistics for Overall Scale Scores**	
Problematic SM use (*mean, SD*)	13.41 (4.98)
Psychological well-being (*mean, SD*)	79.30 (12.24)
Self-esteem (*mean, SD*)	28.33 (6.32)
SM use motivation: Surveillance (*mean, SD*)	19.65 (5.98)
SM use motivation: Network expansion (*mean, SD*)	12.59 (4.20)
SM use motivation: Intrapersonal motive (*mean, SD*)	17.26 (5.18)
SM use motivation: Relationship maintenance (*mean, SD*)	10.35 (2.70)
Positive Affect (*mean, SD*)	31.42 (7.29)
Negative Affect (*mean, SD*)	21.52 (7.52)

*Problematic SM use risk was computed using a strict monothetic cut-off approach, which considered scores ≥ 4 on all six items of the Bergen Social Media Addiction Scale (BSMAS)*.

### Measures

Sociodemographic and social media use characteristics were assessed with questions asking about participants' gender, age, relationship status, preferred social media platform, the number of social media platforms used daily, and whether the platforms were accessed via a smartphone. Additionally, frequency of social media usage was examined using a five-point Likert-scale ranging from very rarely (1) to very often (5), with the following questions: “*How many times during an average day do you use social media?*” and “*How many minutes on average do you spend per session of social media use?*.”

Problematic social media use was measured using the Bergen Social Media Addiction Scale (BSMAS) (Andreassen et al., 2016). The BSMAS is a six-item tool measuring PSMU according to the core components of addiction (i.e., salience, mood modification, tolerance, withdraw symptoms, conflict, and relapse), without a specific timeframe. All six items are answered with a five-point Likert-scale ranging from very rarely (1) to very often (5). Participants were classified as problematic users using a strict monothetic cut-off approach (i.e., scoring four or above on all six items). Total scores on the BSMAS range from 6 to 30, with high scores denoting higher levels of PSMU. The BSMAS has been found to exhibit excellent psychometric properties (Andreassen et al., 2016; Chen et al., 2020). In the present study, the BSMAS exhibited adequate levels of internal consistency (α = 0.83).

Psychological well-being was assessed using the Scales of Psychological Well-Being (SPWB) test (Ryff and Keyes, 1995), which includes 18 items rated on a six-point Likert scale ranging from strongly disagree (1) to strongly agree (6), without a specific timeframe. Total scores can range from 18 to 108, with higher scores suggesting higher levels of psychological well-being. In the present study, the SPWB exhibited high levels of internal consistency (α = 0.83).

Self-esteem was assessed with the 10-item Rosenberg Self-Esteem Scale (RSES) (Rosenberg, 1965) rated on a four-point Likert scale ranging from strongly agree (1) to strongly disagree (4), without a specific timeframe. Total scores can range from 10 to 40, with higher scores indicating greater levels of self-esteem. In the present study, the RSES exhibited excellent levels of internal consistency (α = 0.92).

Social media use motives were assessed using an adapted version of the Motivation for Twitter Use Measure (Lee and Kim, 2014) by replacing the strict term “Twitter” with “social media.” This 14-item measure includes the following four subscales assessing different psychological motivations for using social media websites (without a specific timeframe) in general: *surveillance* (i.e., five items covering the motivation to discover pressing social issues, to obtain various interpretations/explanations on current affairs, to obtain professional knowledge and information, to befriend influential professionals, and to provide useful information to other people; *network expansion* (i.e., three items covering the motivation to provide information about interests to others, to express feelings and thoughts to others, and to befriend people); *intrapersonal motive* (i.e., four items capturing motivational aspects such as to forget the complications of everyday life, to remember what was done, to pass time, and to record everyday life); and *relationship maintenance* (i.e., two items covering the motivation to contact with friends and family, and to provide updates on current life to friends/acquaintances). All the 14 items were rated on a seven-point Likert scale ranging from strongly disagree (1) to strongly agree (7). In the present study, all subscales presented with adequate levels of reliability (surveillance α = 0.75; network expansion α = 0.68; intrapersonal motive α = 0.71; and relationship maintenance α = 0.65).

Positive and negative affect were measured using the 20-item Positive and Negative Affect Schedule (PANAS) (Watson et al., 1988). The PANAS comprises 10 items assessing positive affect and 10 items to assess negative affect without a specific timeframe (i.e., trait-like affect). Participants were requested to indicate their levels of agreement with each item, using a five-point Likert scale ranging from very slightly or not at all (1) to extremely (5). Total scores for both positive and negative affect subscales ranged from 10 to 50, with higher scores indicating higher levels of positive or negative affect. In the present study, the PANAS has exhibited high levels of internal consistency (positive affect α =0.84 and negative affect α = 0.97).

### Statistical Analyses and Data Analytic Strategy

Statistical analyses comprised the following analyses: (i) descriptive statistics of the sample structure, characteristics, and patterns of social media usage; (ii) correlational analysis between the main variables of the study; (iii) independent sample *t*-tests to establish the profile of the sample in terms of PSMU; and (iv) a stepwise multiple linear regression analysis to investigate the role of potential predictors of PSMU (i.e., psychological well-being, self-esteem, social media use motives, and positive and negative affect), while controlling for the effects of age and gender. *R*^2^ and Cohen's *d* (Cohen, 1988) coefficients were estimated for the assessment of goodness of fit of the multiple linear regression model and effect sizes. The analyses align with previous exploratory studies on social media behavior (e.g., Pontes et al., 2018).

Prior to collecting the data, sample size considerations were examined through a power analysis using G^*^Power (version 3.1.9.7) to calculate the minimum sample size needed for the analysis (Faul et al., 2009). This a priori test was based on (i) pre-set power (1-β = 0.95), (ii) medium effect size (*f* ^2^ = 0.15), and (iii) α =0.05, with eight predictors (i.e., psychological well-being, self-esteem, social media use motives, and positive and negative affect) and two sociodemographic and usage variables (age, daily SM use, gender). The results indicated that the required sample size was 172 participants, yielding a power of 0.95.

The procedures for data cleansing included examining the normal distribution, as well as univariate, and multivariate outliers in the dataset. No issues were detected after screening the data, so no further participants were excluded from analyses. The assumptions of the multiple linear regression analysis were examined in order to determine the suitability of the parametric approach. Potential multicollinearity issues were inspected using Variation Inflation Factors (VIF). All VIF values were less than or equal to 1.49, which is well-below the threshold of 10, indicating that there were no multicollinearity issues (Field, 2013). Finally, to minimize Type I errors, Bonferroni correction was applied (Bland and Altman, 1995). The analyses were carried out using IBM SPSS Statistics, Version 26.

## Results

### Descriptive Statistics

All descriptive statistics with means and standard deviations of the variables of interest, can be found in Table 1. Beyond that, all sample characteristics are described in detail in Table 1.

### PSMU Correlates and Profiles

Correlates of PSMU were analyzed with zero-order Pearson correlations (*r*) taking into account all the main variables of the study (see Table 2). Overall, PSMU was positively associated with intrapersonal motive (*r* = 0.51, *p* < 0.01), negative affect (*r* = 0.37), relationship maintenance motive (*r* = 0.31, *p* < 0.01), network expansion motive (*r* = 0.29, *p* < 0.01), and surveillance motive (*r* = 0.25, *p* < 0.01). Furthermore, PSMU was negatively associated with psychological well-being (*r* = −0.30, *p* < 0.01), self-esteem (*r* = −0.27, *p* < 0.01), and positive affect (*r* = −0.08, *p* < 0.05). Correlates for the respondents' age ranged from *r* = −0.29, *p* < 0.01 (problematic SM use) to *r* = 0.19, *p* < 0.01 (self-esteem). Correlates for gender_(ref:1 = female)_ ranged from *r* = −0.10, *p* < 0.01 (network expansion) to *r* = 0.15, *p* < 0.01 (relationship maintenance).

**Table 2:** Correlation matrix across the main variables of the study (*n* = 584).

	***1***	***2***	***3***	***4***	***5***	***6***	***7***	***8***	***9***
1. Problematic social media use	–	−0.30^**^	−0.27^**^	0.25^**^	0.29^**^	0.51^**^	0.31^**^	−0.08^*^	0.37^**^
2. Psychological well-being		–	0.78^**^	−0.04	−0.09^*^	−0.22^**^	0.01	0.45^**^	−0.52^**^
3. Self-esteem			–	−0.09^*^	−0.12^**^	−0.26^**^	−0.08	0.38^**^	−0.55^**^
4. Surveillance				–	0.40^**^	0.21^**^	0.17^**^	0.12^**^	0.13^**^
5. Network expansion					–	0.47^**^	0.49^**^	−0.01	0.13^**^
6. Intrapersonal motive						–	0.50^**^	−0.03	0.28^**^
7. Relationship maintenance							–	0.13^**^	0.10^*^
8. Positive affect								–	−0.08
9. Negative affect									–
Age	−0.29^**^	0.12^**^	0.19^**^	−0.13^**^	−0.10^*^	−0.28^**^	−0.16^**^	−0.09^*^	−0.25^**^
Gender	0.03	0.12^**^	−0.02	−0.09^*^	−0.10^**^	0.07	0.15^**^	−0.01	−0.03

*Social media use motivations subdimensions include surveillance, network expansion, intrapersonal motive, relationship maintenance*;

** *= zero-order Pearson correlation (r) significant at the 0.01 level (two-tailed)*;

* *zero-order Pearson correlation (r) significant at the 0.05 level (two-tailed); Gender ref=1 female*.

Using a strict monothetic cut-off approach, about 6.68% (*n* = 39) of all participants could be classed as problematic users. Furthermore, key differences between problematic and non-problematic users emerged. It was found that problematic users accessed significantly more social media platforms than non-problematic users (Cohen's *d* =0.60). Compared to non-problematic users, problematic users also showed increased levels of negative affect (*d* = 1.08), intrapersonal motive (*d* = 1.07), surveillance (*d* =*0.5*3), network expansion (*d* =*0.4*8), and relationship maintenance (*d* =*0.4*1) motives. Finally, problematic users reported lower levels of psychological well-being (*d* = 0.61) and self-esteem (*d* = *0.5*7), as compared to non-problematic users. Apart from positive affect (*p* = 0.55), all mean differences across the two groups were statistically significant (see Table 3).

**Table 3:** Main differences across social media (SM) users presenting with low and high risk of problematic social media use (*n* = 584).

**Measure**	**Low risk mean (SD)**	**High risk mean (SD)**	**t-statistic**	**df**	**Mean differences**	**CI lower**	**CI upper**	**Cohen's d**
Number of SM platforms used	2.15 (1.19)	2.90 (1.29)	−3.48	42.77	−0.74	−1.17	−0.31	0.60
Psychological well-being	4.43 (0.66)	4.00 (0.73)	3.64	42.66	0.44	0.20	0.68	0.61
Self-esteem	28.59 (6.19)	24.74 (7.19)	3.25	42.12	3.84	1.46	6.23	0.57
Surveillance	19.43 (5.90)	22.69 (6.22)	−3.17	43.05	−3.26	−5.33	−1.19	0.53
Network expansion	12.46 (4.19)	14.41 (3.91)	−3.00	44.47	−1.96	−3.27	−0.64	0.48
Intrapersonal motive	16.92 (5.07)	22.03 (4.38)	−6.95	45.62	−5.11	−6.58	−3.63	1.07
Relationship maintenance	10.28 (2.69)	11.38 (2.59)	−2.56	44.10	−1.10	−1.97	−0.24	0.41
Positive affect	31.52 (7.22)	30.03 (8.18)	1.11	42.35	1.49	−1.23	4.21	0.19
Negative affect	20.96 (7.16)	29.31 (8.22)	−6.17	42.23	−8.34	−11.07	−5.62	1.08

*Results are statistically significant after applying Bonferroni correction to account for potential Type I error (i.e., p < 0.006); SD, standard deviation; df, degrees of freedom; CI, confidence interval*.

### Predictors of PSMU

A stepwise multiple linear regression was carried out to explore the predictors of PSMU using the main variables of the study. The final model estimated in the fifth step (see Table 4) included intrapersonal motive (β = 0.37, *t* = 10.67), negative affect (β = 0.22, *t* = 6.66), daily social media use (β = 0.18, *t* = 5.37), surveillance motive (β = 0.12, *t* = 3.35), and positive affect (β = −0.09, *t* = −2.80) as significant predictors of PSMU, contributing ~37.3% of the total variance in PSMU scores. Among all predictors, intrapersonal motive (β = 0.37) provided the strongest predictive contribution (Δ*R*^2^ = 0.009; Δ*F* [1, 577] = 7.88, *p* = 0.005). The final model excluded the following predictors due to low, or non-statistically significant, predictive power: psychological well-being (*p* = 0.10), self-esteem (*p* = 0.51), network expansion motive (*p* = 0.82), and relationship maintenance motive (*p* = 0.06). Finally, the control variables age and gender emerged as non-significant predictors of PSMU (*p* > 0.05).

Table 4The relationship between problematic social media use and key related predictors.

**Table:** 

***Predictors***	***Step 1***			***Step 2***			***Step 3***			***Step 4***			***Step 5***		
	***B***	***SE***	**β**	***B***	***SE***	**β**	***B***	***SE***	**β**	***B***	***SE***	**β**	***B***	***SE***	**β**
Intrapersonal motive	0.50	0.03	0.51^***^	0.43	0.03	0.43^***^	0.39	0.03	0.40^***^	0.37	0.04	0.38^***^	0.37	0.03	0.38^***^
Negative affect				0.16	0.02	0.16^***^	0.16	0.02	0.24^***^	0.16	0.02	0.23^***^	0.15	0.02	0.22^***^
Daily social media use							0.79	0.14	0.19^***^	0.74	0.14	0.18^***^	0.77	0.14	0.18^***^
Surveillance										0.09	0.03	0.10^**^	0.10	0.03	0.12^**^
Positive affect													−0.06	0.02	−0.09^**^

**Table:** 

*Model summary*					
Variance explained by the model	*R*^2^ =0.263 (26.3%)	*R*^2^ =0.320 (32%)	*R*^2^ = 0.354 (35.4%)	*R*^2^ = 0.364 (36.4%)	*R*^2^ = 0.373 (37.3%)
Change in variance by increasing step		Δ*R*^2^ = 0.057 (57%)	Δ*R*^2^ = 0.034 (34%)	Δ*R*^2^ = 0.010 (10%)	Δ*R*^2^ = 0.009 (0.9%)
Statistical significance of the model	*F*_(1, 581)_ = 207.66^***^	*F*_(2, 580)_ = 136.36^***^	*F*_(3, 579)_ = 105.79^***^	*F*_(4, 578)_ = 82.72^***^	*F*_(5, 577)_ = 68.54^***^
Statistical significance of steps		Δ*F*_(1, 580)_ = 48.20^***^	Δ*F*_(1, 579)_ = 30.69^***^	Δ*F*_(1, 578)_ = 9.08^**^	Δ*F*_(1, 577)_ = 7.88^**^

*** *p < 0.001*;

** *p < 0.05*.

*The final model, i.e., Step 5 excluded the following variables due to low or non-significant predictive power: age and gender (control variables), psychological well-being, self-esteem, network expansion, and relationship maintenance. B, unstandardized regression coefficient; SE, standard error; β, standardized regression coefficient; R^2^, R square; ΔR^2^, R square change*.

## Discussion

This study contributes to the growing field of PSMU by investigating the role of key correlates and predictors of PSMU, broadly defined. This is an important contribution to the field given that a relatively large amount of previous research has focused on problematic Facebook use specifically, and exclusively. Analyses of the data in the present study revealed that intrapersonal motive, negative affect, relationship maintenance, and psychological well-being were the strongest correlates of PSMU. Furthermore, intrapersonal motive explained about 26% of the total variance in PSMU, followed by negative affect (14%), and relationship maintenance (10%), respectively. Interestingly, broad social media use motives produced strong effects, which is in line with findings from uses and gratification theory (Blumler and Katz, 2020), for instance showing that among others, social motives play an important role in understanding social media use [both to predict intention to use (see Hossain, 2019) and continuance usage Hsiao et al. (2016)]. A recent work by Sariyska et al. (2019) also found support for the relevance of investigating the affiliative motive in the context of Facebook use, but findings might be also dependent on the cultural context. In general, these observations also fit with empirical findings from personality psychology, demonstrating that social media users tend to be slightly more extraverted than non-users (Marengo et al., 2020; Sindermann et al., 2020) and it is well-known that extraverts have a stronger urge to socially interact with other persons. Note that much research on diverse social media use motives has been conducted in the realm of normal or healthy social media use.

As one can see, previous research has considered the relationships between social media use motives and specific factors as described above, such as affiliation and communication confidence (Lee and Kim, 2014). Nevertheless, we believe the present study is among the first, to the authors' knowledge, to explore the relationship between broad social media use motives and PSMU. Moreover, these findings align with previous literature on social media use by supporting the relationship between PSMU with psychological well-being (Oh et al., 2014), self-esteem (Mei et al., 2016), and psychological affect (Caplan, 2002, 2010).

The findings obtained further indicated that about 6.68% of the sample recruited could be classed as problematic users due to their high-risk profile of PSMU. This result aligns with previous research reporting that social media related problems due to PSMU may range from 1.6% (Alabi, 2013) to 18% (Moghavvemi et al., 2017). Although epidemiological data on PSMU has been previously reported, additional research on this emerging phenomenon is warranted before any formal psychiatric recognition and further generalizations to the broader population can be made.

Notwithstanding this, the findings obtained in this study suggest that problematic users used significantly more social media platforms, and presented with increased levels of negative affect, intrapersonal, surveillance, network expansion, and relationship maintenance motives compared to non-problematic users. Furthermore, problematic users also presented with decreased levels of psychological well-being and self-esteem in comparison to non-problematic users. These findings contribute to the growing body of evidence linking PSMU with maladaptive cognition, increased psychiatric distress (Pontes et al., 2018), and decreased well-being (Kross et al., 2013; Satici and Uysal, 2015; Błachnio et al., 2016b).

In terms of key PSMU predictors, the most relevant factors related to intrapersonal motive, followed by negative affect, daily social media use, surveillance motive, and positive affect, which explained about 37.3% of the total variance in PSMU. This finding aligns with past research reporting that key predictive factors associated with PSMU are over and above the influence of commonly related sociodemographic variables such as gender and age (Pontes et al., 2018).

The intricate connection between intrapersonal motive for using social media and PSMU should be further investigated beyond the linear relationship reported in this study. Intrapersonal motives comprise self-directed emotions associated with self-esteem, guilt, and shame (Weiner, 2001). This finding supports the notion that individuals using social media in an attempt to better understand themselves and their environment are likely to exhibit greater levels of PSMU, a contention that aligns with emerging research showing that trait emotional intelligence may be thought of as a risk factor for PSMU (Kircaburun et al., 2019; Süral et al., 2019). This idea needs to be supplemented by recent notions to better understand the detrimental aspects of social media use by distinguishing between active and passive use, whereas passively browsing seems to be associated with the aspects of overuse and negative affect (e.g., Escobar-Viera et al., 2018).

The current findings also indicated that PSMU may be explained by other social media use motives since they contributed to predicting PSMU in the present sample. Furthermore, the results indicated that self-esteem and the use of multiple social media platforms were linked to PSMU, which may highlight the users' need to improve their levels of self-image and self-esteem, particularly among problematic users (Błachnio et al., 2016a). In this context, it is worth mentioning the process of upward social comparison, which can be triggered by comparing the number of “Likes” with others or by comparing life styles (Vogel et al., 2014). Taken together, these findings may provide useful information with potential clinical implications about the way individuals use social media in the context of the coronavirus (COVID-19) pandemic as recent studies have reported that increased exposure to disaster-related information on social media may trigger negative affect, which can elicit mental health disorders (Zhao and Zhou, 2020), a contention that is aligned with the findings obtained since negative affect was a strong predictor of PSMU.

Despite its contributions, the current study presents with several potential limitations. Although this study found consistent associations between PSMU and psychological well-being, self-esteem, negative affect, and social media use motives, its cross-sectional nature does not allow for causal inferences to be made. Future research should adopt a longitudinal design to further examine the temporal role of key mechanisms underlying PSMU. Another potential limitation in the present study relates to the self-report methodology used, which is likely to have generated well-known biases and unreliable estimations of participants' objective social media use (Rozgonjuk et al., 2020c). Consequently, it is of high interest to also record behavior directly due to time distortions participants experience while using technology (e.g., Lin et al., 2015; Montag et al., 2015).

Importantly, the present study drew its conclusions on a relatively small sample that was sampled through convenience and self-selection sampling, further limiting the scope of the findings as any research question addressed with convenience sampling is limited to the sample itself (Bornstein et al., 2013). We also acknowledge that the *ad-hoc* cut-off approach of the BSMAS, despite being strict, lacks information about its diagnostic accuracy (e.g., sensitivity and specificity) using clinically diagnosed samples as a gold standard. Finally, another potential limitation related to the present study relates to the relatively limited number of possible social media use motives that have been investigated, therefore it is likely that different results would have emerged in light of different psychological motives.

Notwithstanding these potential limitations, this study helps advancing the field of PSMU by further elucidating its key correlates and predictors in terms of psychological well-being, self-esteem, social media use motives, and psychological affect. In conclusion, we found that PSMU was positively associated with intrapersonal motive, negative affect, relationship maintenance motive, network expansion motive, and surveillance motive and negatively associated with psychological well-being, self-esteem, and positive affect. Finally, key predictors of PSMU were related to intrapersonal motive, negative affect, daily social media use, surveillance motive, and positive affect.

## Data Availability Statement

The dataset presented in this article is not readily available due to limitations in the confidentiality as per the SREC's recommendations. Requests to access the datasets should be directed to Dr. Bruno Schivinski, bruno.schivinski@rmit.edu.au.

## Ethics Statement

The studies involving human participants were reviewed and approved by the School of Social Sciences Research Ethics Committee (SREC) Nottingham Trent University. The patients/participants provided their written informed consent to participate in this study.

## Author Contributions

BS has performed all final statistical analyses of the study and helped drafting, revising, and reviewing the manuscript. MB-W, JS, and CM have all contributed extensively to revising the manuscript and writing its final version. ES was involved in the design of the study, preliminary statistical analyses, and recruitment of participants in addition to helping draft the manuscript. HP has designed the study, assisted with the preliminary statistical analyses, drafting, reviewing, and revision of all versions of the manuscript.

## Conflict of Interest

The authors declare that the research was conducted in the absence of any commercial or financial relationships that could be construed as a potential conflict of interest.
